# Renal actinomycosis presenting as a suppurated solitary cyst

**DOI:** 10.4103/0970-1591.42631

**Published:** 2008

**Authors:** Ioannis Efthimiou, Charalampos Mamoulakis, Konstantina Petraki, Ioannis Zorzos

**Affiliations:** Department of Urology, Evangelismos General Hospital, Athens, Greece; 1 Department of Pathology, Evangelismos General Hospital, Athens, Greece

**Keywords:** Actinomycosis, kidney, cyst

## Abstract

We report the case of a middle-aged female with a solitary renal cyst suppurated by *Actinomyces israeli*. The patient was treated successfully by emergency nephrectomy. Based on a detailed negative history of inciting events and predisposing factors for actinomycosis, negative imaging modalities for extra-renal organ implication and no involvement of the surrounding renal parenchyma, the case is considered as primary renal actinomycosis affecting a solitary cyst of the kidney. This is the first such case reported in the international literature.

## INTRODUCTION

Actinomycosis is an uncommon infection, which generally appears in the cervicofascial, thoracic and abdominal regions. The responsible microorganism is *Actinomyces israeli*, a gram-positive anaerobic bacterium. Renal actinomycosis is very rare and it often mimics neoplasia. It usually affects the renal parenchyma and the adjacent tissues. Diagnosis is established by culture but definite diagnosis is made by histological identification of sulphur granules. Conservative medical treatment with preservation of the kidney has been described.[1] To the best of our knowledge, a case of primary actinomycosis affecting a solitary renal cyst (SRC) without involving the renal parenchyma has never been reported.

## CASE REPORT

A 50-year-old, previously healthy female presented with a three-week history of high fever, rigor, malaise, lower urinary tract symptoms, weight loss and left loin pain with localized tenderness on physical examination. Laboratory tests revealed excessive leucocytosis (21.530/mm^3^), thrombocytosis (5 × 10^5^/mm^3^), elevated erythrocyte sedimentation rate (85mm/h), abnormal liver chemistry (AST:69U/L, ALT:93U/L, ALP:122U/L), pyuria and microhematuria. Abdominal ultrasound scan revealed an 8 × 8 cm centrally-located, left renal mass with a central liquid portion and a thick surrounding wall.

**Figure 1:**
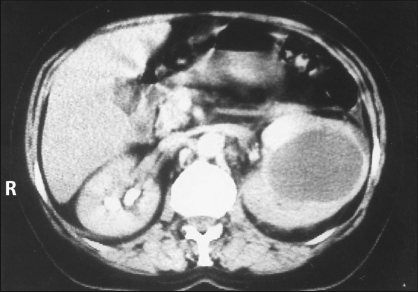
Upper abdominal CT scan after intravenous contrast medium administration. An enlarged left kidney with a solitary cyst was observed with homogeneous and low-density content. Cystic wall enhancement was not observed in the excretory phase

Further evaluation with computed tomography (CT) scan of the abdomen revealed an enlarged left kidney carrying a 5 × 9 cm well-delineated cystic lesion of homogeneous-low density content (Hounsfield units: zero to three), surrounded by a thickened outer wall, which occupied the upper-middle renal part in close proximity to the pelvis [Figure 1]. Cystic wall enhancement was lacking after intravenous administration of contrast medium during the excretory phase.

Differential diagnosis included unilocular cystic carcinoma associated with paraneoplastic syndrome, abscess, complicated cyst and hydatid disease. Percutaneous drainage was avoided due to the possibility of seeding in case of a carcinoma or anaphylaxis in case of a hydatid cyst rupture. Despite rigorous treatment with cefuroxime and amikacin, the patient's condition deteriorated with the advent of early signs of sepsis three days later. Emergency renal exposure via a left flank extra-peritoneal approach disclosed a large, grossly inflamed kidney carrying a space-occupying lesion with irregular surface, favoring nephrectomy. The postoperative course was uneventful.

**Figure 2:**
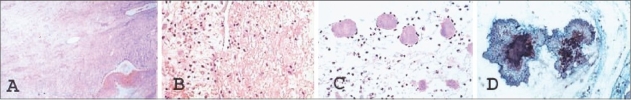
(A) Section of the cystic wall indicating a simple renal cyst (Hematoxylin-Eosin ×20). (B) Abscess formation in the cavity of the cyst (Hematoxylin-Eosin ×100). (C) Grains of *Actinomyces israeli* surrounded by pus cells (Hematoxylin-Eosin ×100). (D) Grains of *Actinomyces israeli* stained by Grocott's stain (×200)

Gross inspection of the specimen revealed a solitary pelvic cyst, filled with hoary-rotten material, which displaced the surrounding parenchyma completely and was surrounded by a renal hematoma. Renal pelvis was slightly dilated. Microscopic examination showed a simple renal cyst surrounded by focal spotty hemorrhages, focal hyperplasia, reactive atypia, sclerotic changes of the glomeruli and chronic active tubulointerstitial nephritis. No elements of a neoplastic process were detected. Examination of the pus was negative for Koch's bacilli or other bacteria but revealed *Actinomyces israeli* [Figure 2].

A postoperative CT scan ruled out thoracic actinomycosis. The patient received complementary treatment with amoxicillin for three months but she was lost during the follow-up period.

## DISCUSSION

*Actinomyces israeli* is a gram-positive anaerobic bacterium, normally found in the oral cavity and the gastrointestinal tract. It usually becomes pathogenic only in the presence of pathologically affected/necrotic tissue. The cervicofascial region is involved in about 60% of the cases. Dental caries represent a potential source.

Actinomycosis of the genitourinary system is usually secondary to direct extension from contiguous abdominal sources or subsequent retroperitoneal spread. Hematogenous spread occurs rarely.[2] Previous surgery, dental extraction, trauma and intrauterine contraceptive device use represent predisposing factors of the disease. Renal involvement may mimic neoplasia.[3] The disease presents clinically with general symptoms of chronic disease such as low-grade fever, loin pain and weight loss, although high-grade fever, sepsis and death have been reported.[2,3] Actinomycosis of the kidney may present as: renal abscess, abscess forming pyelonephritis, pyonephrosis with renal calculosis and necrotizing papilllitis. Two types of CT patterns have been described in renal actinomycosis:[4] a) solid masses with areas of low attenuation and variable contrast enhancement and b) cystic masses with thick enhancing walls.

This is the first report of an SRC suppurated by *Actinomyces israeli.* Our patient had no obvious inciting events such as a history of recent or remote bowel surgery (e.g. perforated acute appendicitis, perforated colonic diverticulitis following trauma to the abdomen), ingestion of foreign bodies (e.g. chicken or fish bones) or a past history of intrauterine contraceptive device use.

Although renal actinomycosis usually presents as an infiltrative mass lesion, an interesting point is that *Actinomyces israeli* was restricted within the cyst without involving the renal parenchyma. Usually, histology reveals yellow-grey nodules with microabscesses which contain the characteristic lobular deep purple micro-organism colonies (sulphur granules).[3] Thoracic, lower abdominal and pelvic CT scans were normal. Therefore, based on these facts, SRC actinomycosis is considered to be primary in this case.

Suppuration of an SRC is generally rare. Infection occurs primarily via the ascending route. Hematogenous remote seeding has also been described. Infection most commonly involves gram-negative organisms. Gram-positive (Staphylococcus) or other organisms are extremely rare. A CT scan may set the diagnosis of a suppurated renal cyst by indicating wall thickening and enhancement. However, in our case CT scan findings were not suggestive of suppuration, since Hounsfield units were those of water density and mural enhancement was lacking. Furthermore, premorbid CT scans were not available for review. In our opinion, CT scan helps in the diagnosis of a suppurated SRC mainly in cases with baseline previous scans available, where the size and density change of the cyst can be compared.

Planning of the appropriate management (percutaneous aspiration, cyst excision or nephrectomy) presupposes an early and accurate diagnosis, which is not always that easy. Conservative management of renal actinomycosis with high doses of penicillin is an accepted alternative considering a high degree of suspicion and an early diagnosis with fine needle aspiration.[1] However, CT findings could not exclude carcinoma/hydatid cyst, precluding drainage. Although *Actinomyces* species appear to be susceptible to a wide range of beta-lactam agents which, combined with beta-lactamase inhibitors are regarded as agents of first choice, it is well known that infected simple SRCs are highly resistant to antimicrobial chemotherapy due to poor antibiotic penetration.[5] Therefore, it is unknown whether in our case high doses of penicillin alone could have been curative considering the septic status of the patient and the pharmacokinetic properties of beta-lactam agents in simple SRCs.
